# Author Correction: De novo emergence of adaptive membrane proteins from thymine-rich genomic sequences

**DOI:** 10.1038/s41467-020-20609-y

**Published:** 2021-01-04

**Authors:** Nikolaos Vakirlis, Omer Acar, Brian Hsu, Nelson Castilho Coelho, S. Branden Van Oss, Aaron Wacholder, Kate Medetgul-Ernar, Ray W. Bowman, Cameron P. Hines, John Iannotta, Saurin Bipin Parikh, Aoife McLysaght, Carlos J. Camacho, Allyson F. O’Donnell, Trey Ideker, Anne-Ruxandra Carvunis

**Affiliations:** 1 Smurfit Institute of Genetics, Trinity College Dublin, University of Dublin, Dublin 2, Ireland; 2 Department of Computational and Systems Biology, School of Medicine, University of Pittsburgh, Pittsburgh, PA 15213 USA; 3 Pittsburgh Center for Evolutionary Biology and Medicine, School of Medicine, University of Pittsburgh, Pittsburgh, PA 15213 USA; 4 Department of Medicine, Division of Medical Genetics, University of California San Diego, La Jolla, CA 92093 USA; 5 Department of Biological Sciences, University of Pittsburgh, Pittsburgh, PA 15260 USA

Correction to: *Nature Communications* 10.1038/s41467-020-14500-z, published online 7 February 2020.

The original version of this Article contained an error in the Competing interests section, which incorrectly omitted “T.I. is a co-founder of Data4Cure and has an equity interest. T.I. is on the Scientific Advisory Board of Ideaya BioSciences, Inc., has an equity interest, and receives income. The terms of these arrangements have been reviewed and approved by the University of California San Diego in accordance with its conflict of interest policies”. This has been corrected in both the PDF and HTML versions of the Article.

