# Supplementary material for: De novo emergence of adaptive membrane proteins from thymine-rich genomic sequences
Source: Nat Commun. 2020 Feb 7;11:781. doi: 10.1038/s41467-020-14500-z (PMC7005711; doi:10.1038/s41467-020-14500-z)
Supplement: Supplementary file 1 — Supplementary Information [file 41467_2020_14500_MOESM1_ESM.pdf]

SUPPLEMENTARY INFORMATION

De novo emergence of adaptive membrane proteins from thymine-rich genomic sequences

Vakirlis *et al.*

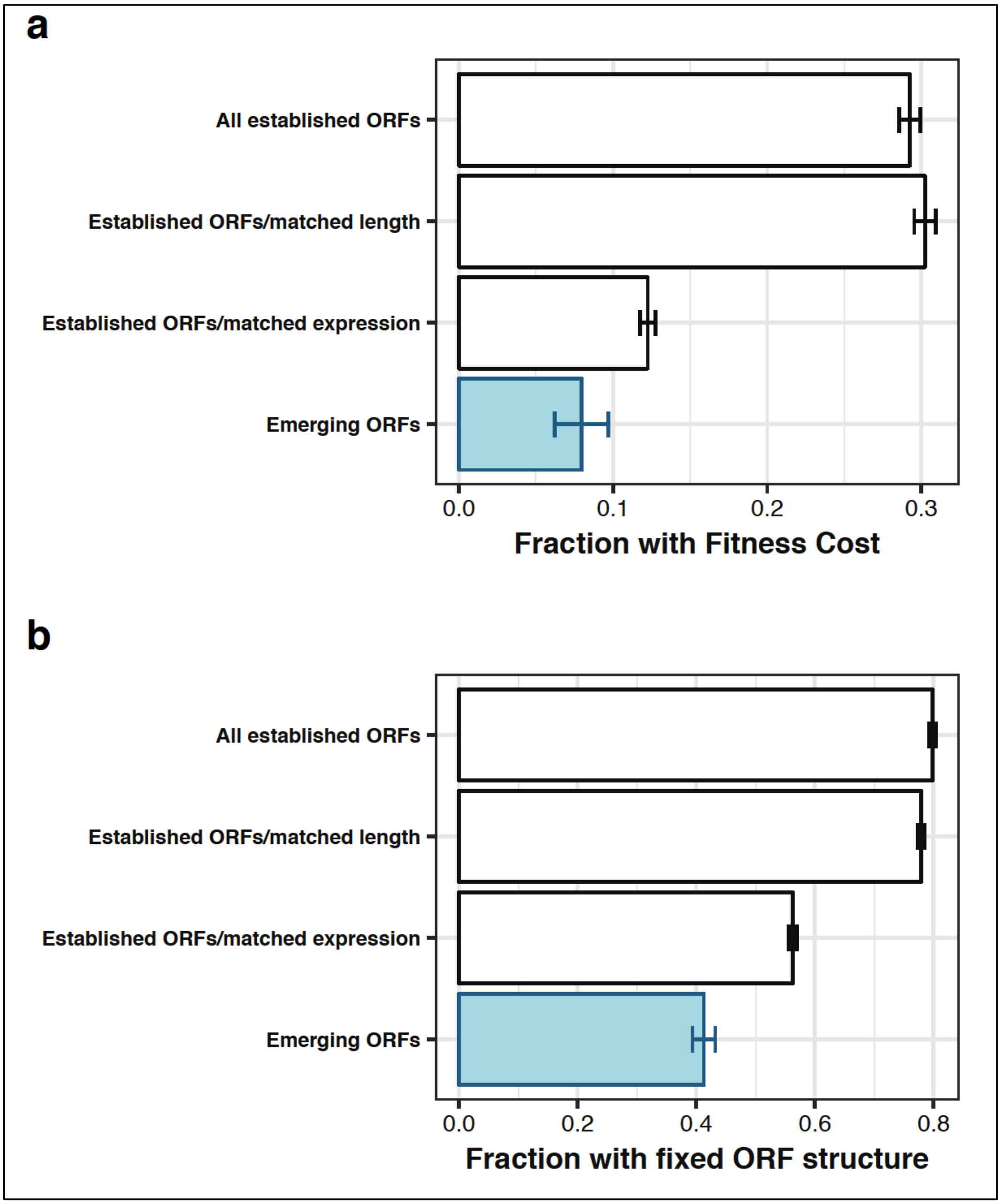

Supplementary Fig. 1. Comparing the fitness impact of disrupting emerging and established ORFs with matched expression levels and lengths distributions.

Established ORFs with length and expression level distributions matched to those of emerging ORFs were randomly sampled with replacement among the set of all established ORFs (**Methods**).

a. Fraction of ORFs for which experimental disruption leads to colonies with fitness <0.9, as in **Fig. 2a**. Error bars: standard error of the proportion.

b. Fraction of ORFs with fixed ORF structure in 90% of *S. cerevisiae* isolates analyzed in **Fig. 2b**. Error bars: standard error of the proportion.

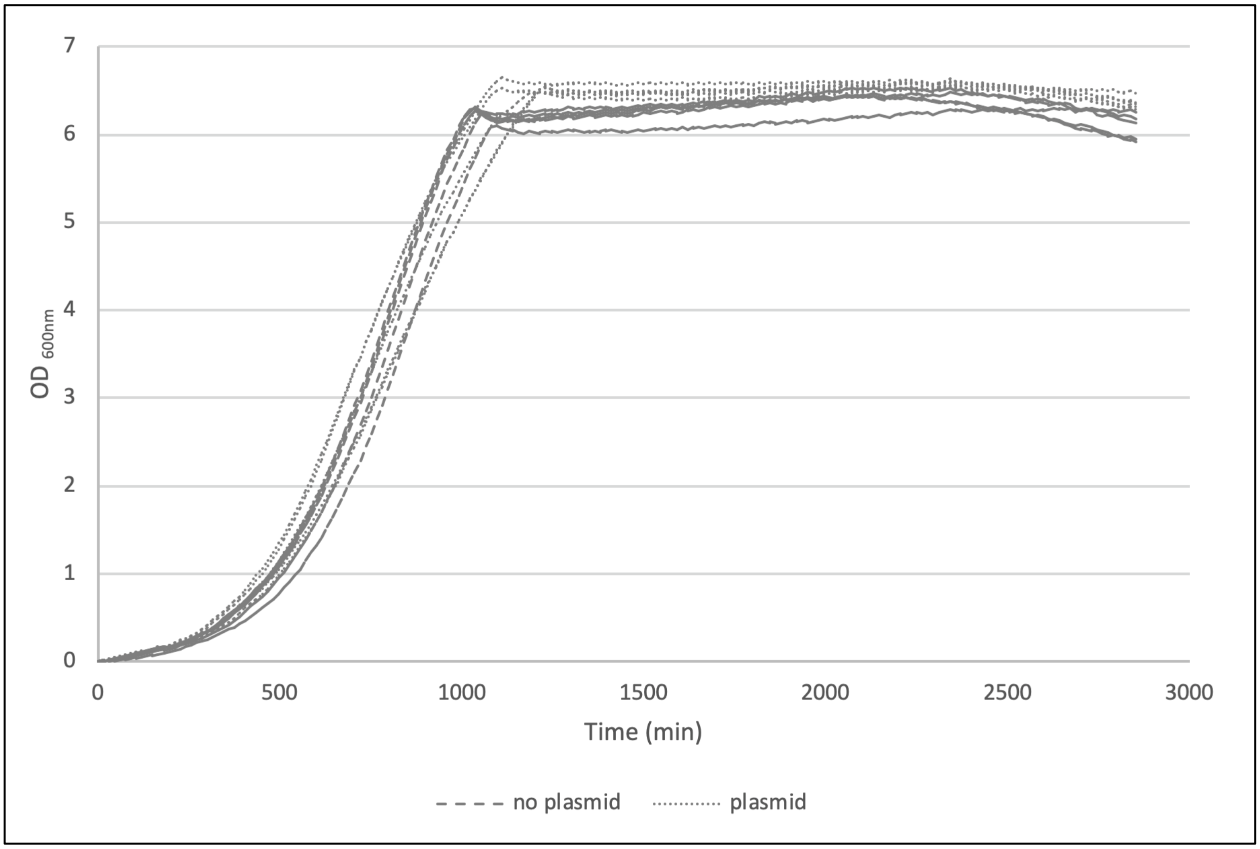

Supplementary Fig. 2. No detectable growth defect in the neutral reference strain.

The reference strain was tested for growth defect by using the barcoded haploid yeast overexpression strain (**Supplementary Table 2**) transformed with expression vector (pBY011) (“plasmid”) and the same strain without vector (“no plasmid”). The strains were grown in SC-URA+GAL+G418 and SC+URA+GAL+G418, respectively. (**Supplementary Table 1**). A flat bottom 96-well plate was filled with replicates of diluted pre-cultures (125µl at OD_{600nm} 0.08) and used to collect OD_{600nm} points every 15 min for 48h using a plate reader (SpectraMax M2, Molecular Devices). For each strain, 5 biological replicates represented by 4 technical replicates each were included. Raw data were blank corrected to the respective media, path length corrected and normalized to time zero.

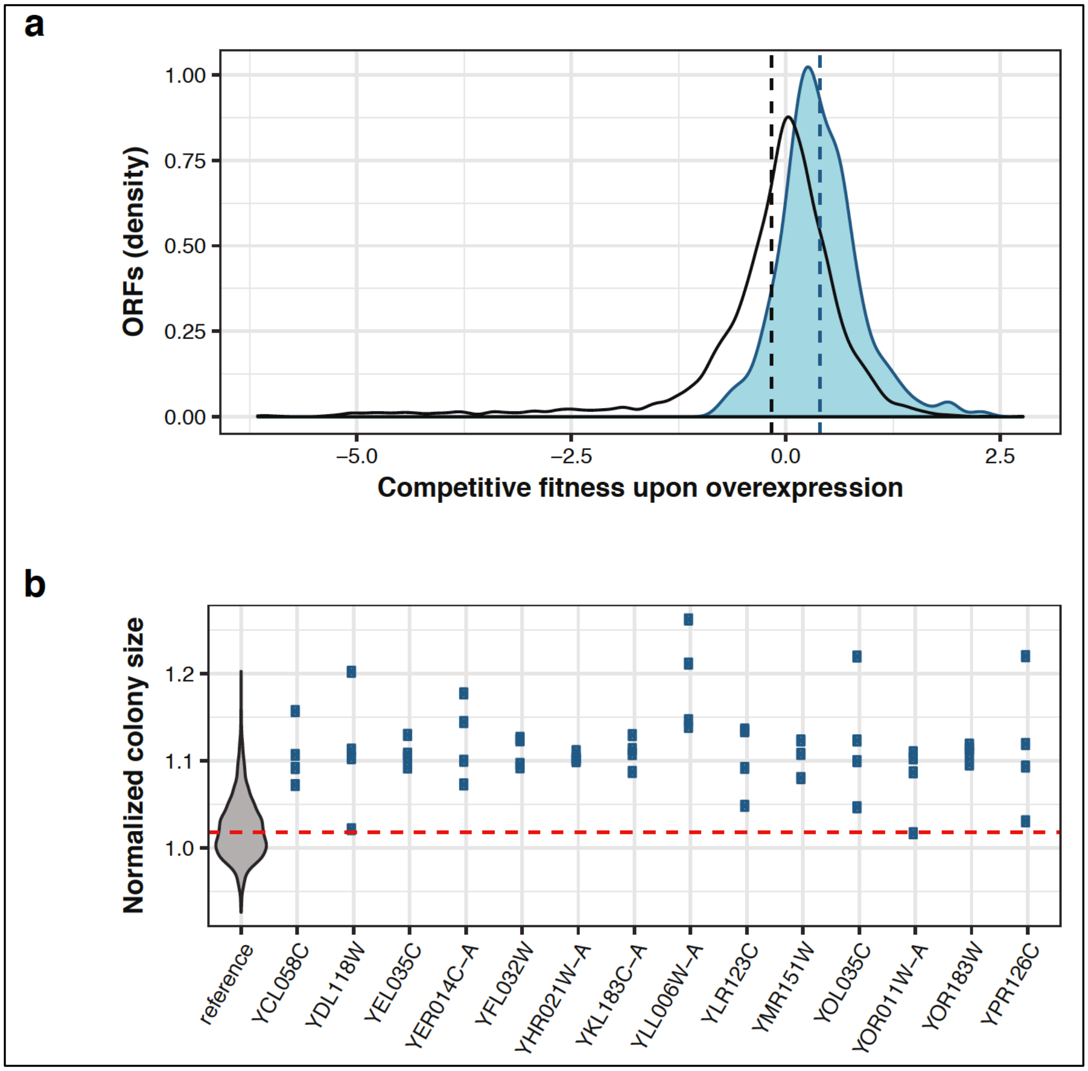

Supplementary Fig. 3: Overexpression of emerging ORFs can provide fitness benefits.

a. Emerging ORFs display higher competitive fitness when overexpressed than established ORFs (Mann-Whitney U test *P* = 5.5x10^{-32}). Density distributions for emerging (blue) and established (black) ORFs competitive fitness measurements in complete media after 20 generations, as measured and quantified through barcode signal intensity by[1]. Vertical dashed lines represent group means. Note that this experimental design did not allow for direct comparison with the fitness of a reference strain.

b. Distribution of the normalized sizes of individual replicate colonies for the reference strain (grey violin plot on the left) and for each emerging ORFs identified as statistically increasing fitness relative to the reference in complete media (see **Fig. 3b**). Red dashed line represents the median normalized colony size of the reference strain. Each of the 14 emerging ORFs represented here present distributions of normalized colony sizes that are incompatible with the null hypothesis according to which they could have been randomly picked from the reference distribution (and hence were detected by our pipeline as showing increased relative fitness). Colony sizes of each replicate of all strains included in this screen are presented in **Supplementary Data 2**.

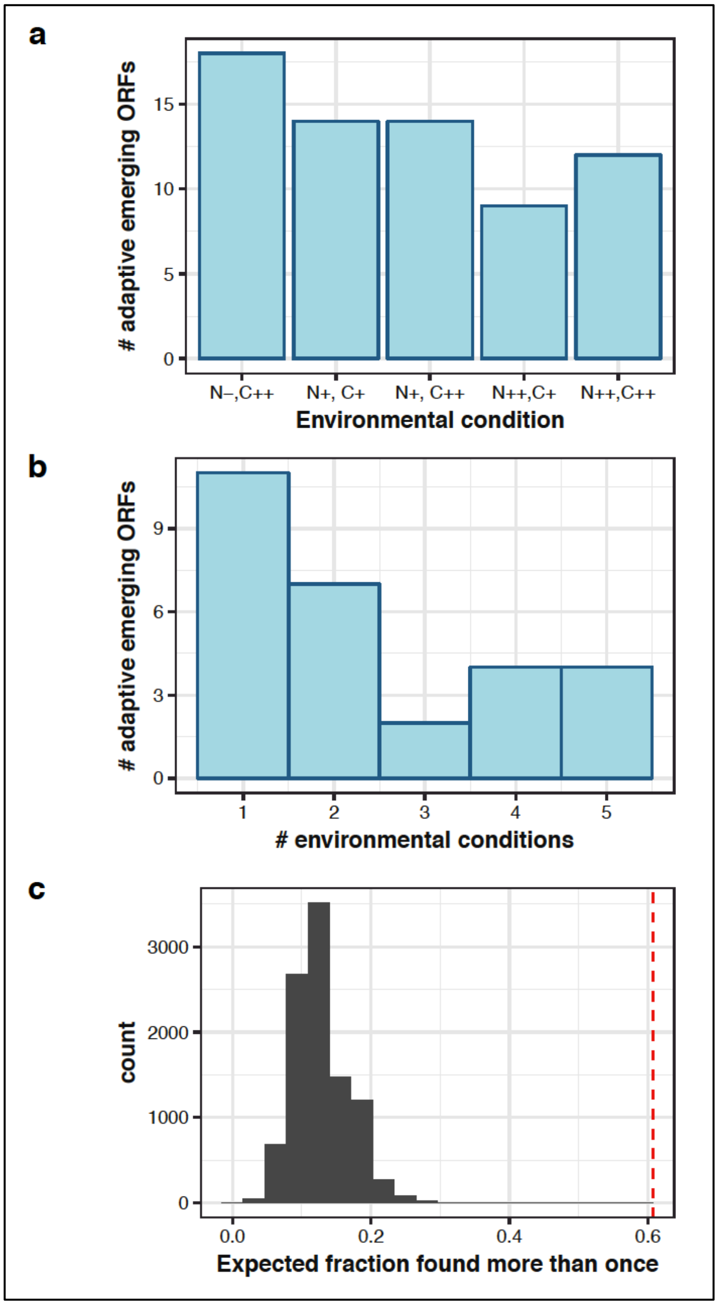

Supplementary Fig. 4. Emerging ORFs can increase relative fitness across environments.

a. Number of emerging ORFs detected as increasing relative fitness in 5 environmental conditions. See **Supplementary Table 1** for environment composition details and **Fig. 3d** for comparison with established ORFs.

b. Most emerging ORFs found to increase relative fitness in at least one environment are also found to increase fitness in another environment. Of 28 adaptive emerging ORFs (**Fig. 3e**), 11 were detected in a single environment and 17 (60%) were found more than once.

c. Expected fraction of emerging ORFs that would be found to increase relative fitness in more than one environment under a stochastic null model where ORFs are drawn randomly from the set of emerging ORFs never found deleterious in our experiments, to simulate the distribution of (**a**) (five draws with replacement of the same number of elements as the real data). 10,000 simulations were run and the observed proportion (**b**, 60%) was never observed.

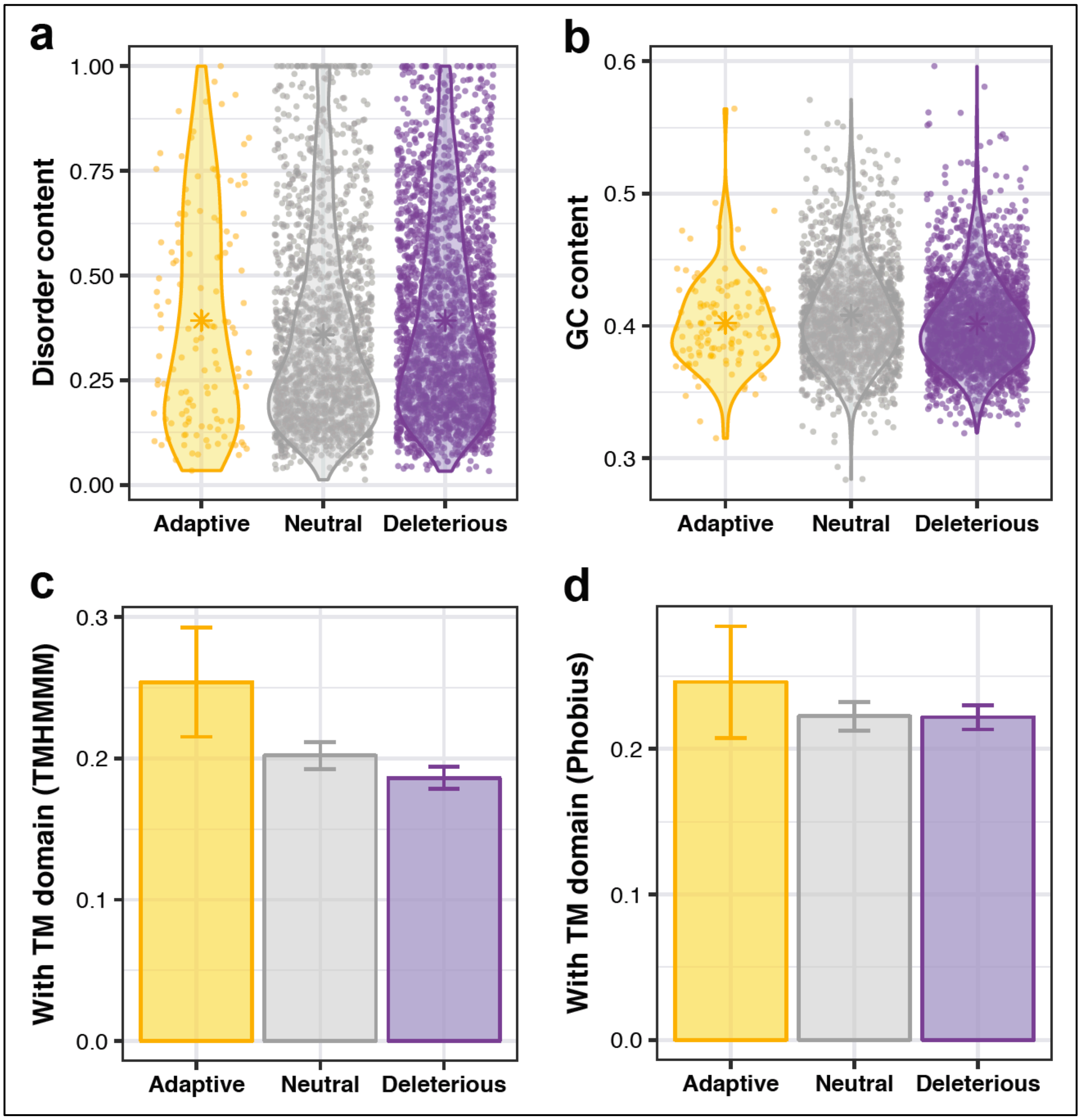

Supplementary Fig. 5. No clear beneficial capacities in established ORFs.

The analyses presented in **Fig. 4** were repeated on the group of established ORFs. Disorder content (**a**) is statistically indistinguishable between adaptive and neutral or deleterious established ORFs (Mann-Whitney U test P>0.3 in both cases). GC content (**b**) is also indistinguishable between adaptive and neutral or deleterious established ORFs (Mann-Whitney U test P>0.1 in both cases). See also **Supplementary Fig. 6**. Error bars in (**c**) and (**d**): standard error of the proportion. Stars represent averages of the populations.

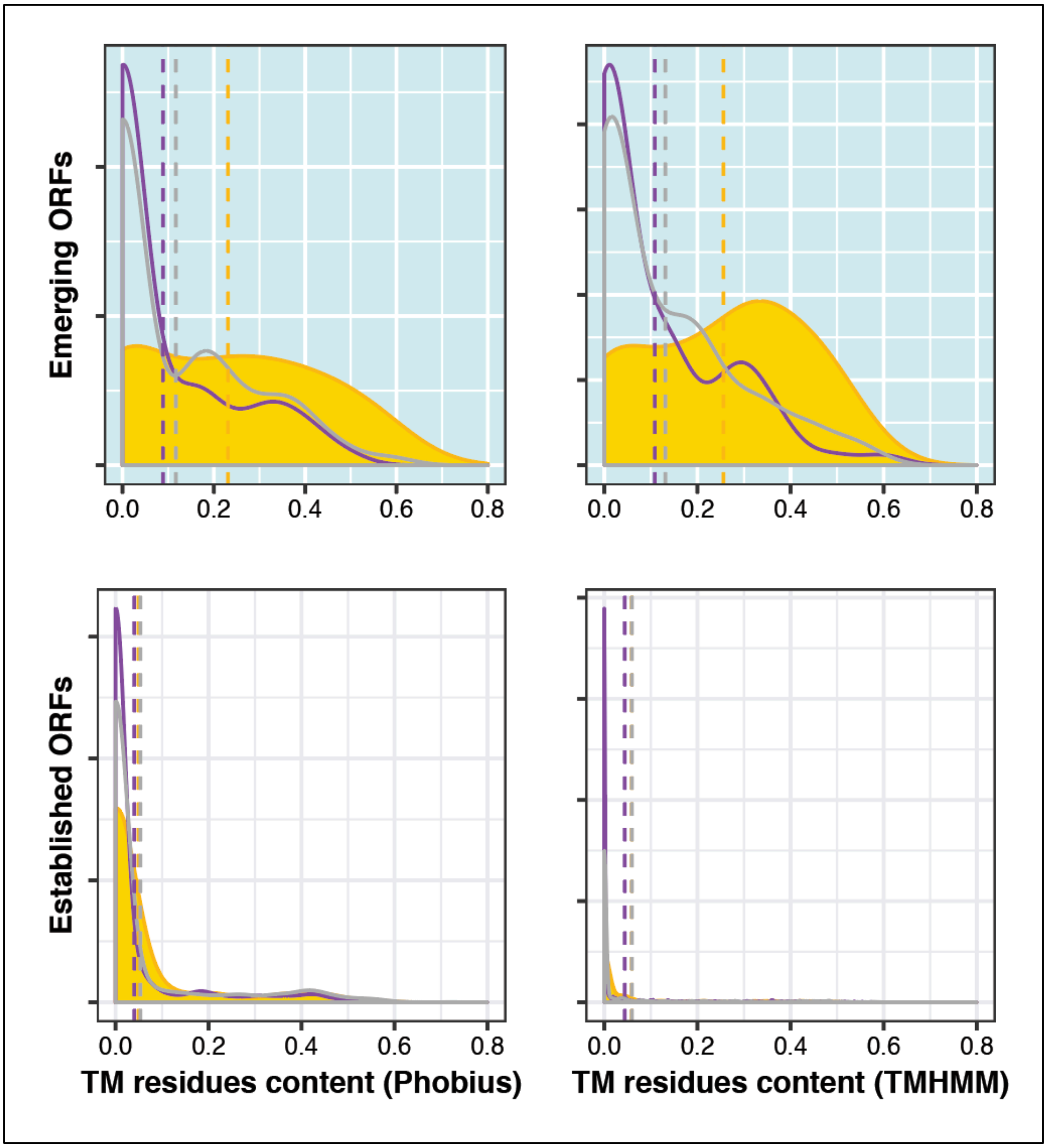

Supplementary Fig. 6. Distribution of TM residue content.

Two prediction methods are compared: Phobius (left column) and TMHMM (right column). Two ORF classes are compared: emerging (top row) and established (bottom row) ORF. In each case, the distribution (density plot) of TM residue content (fraction of amino acids predicted as TM over length of the ORF) is shown for ORFs classified as adaptive (gold), deleterious (purple) and neutral (gray). Vertical dashed lines correspond to the mean of the distribution of the respective color.

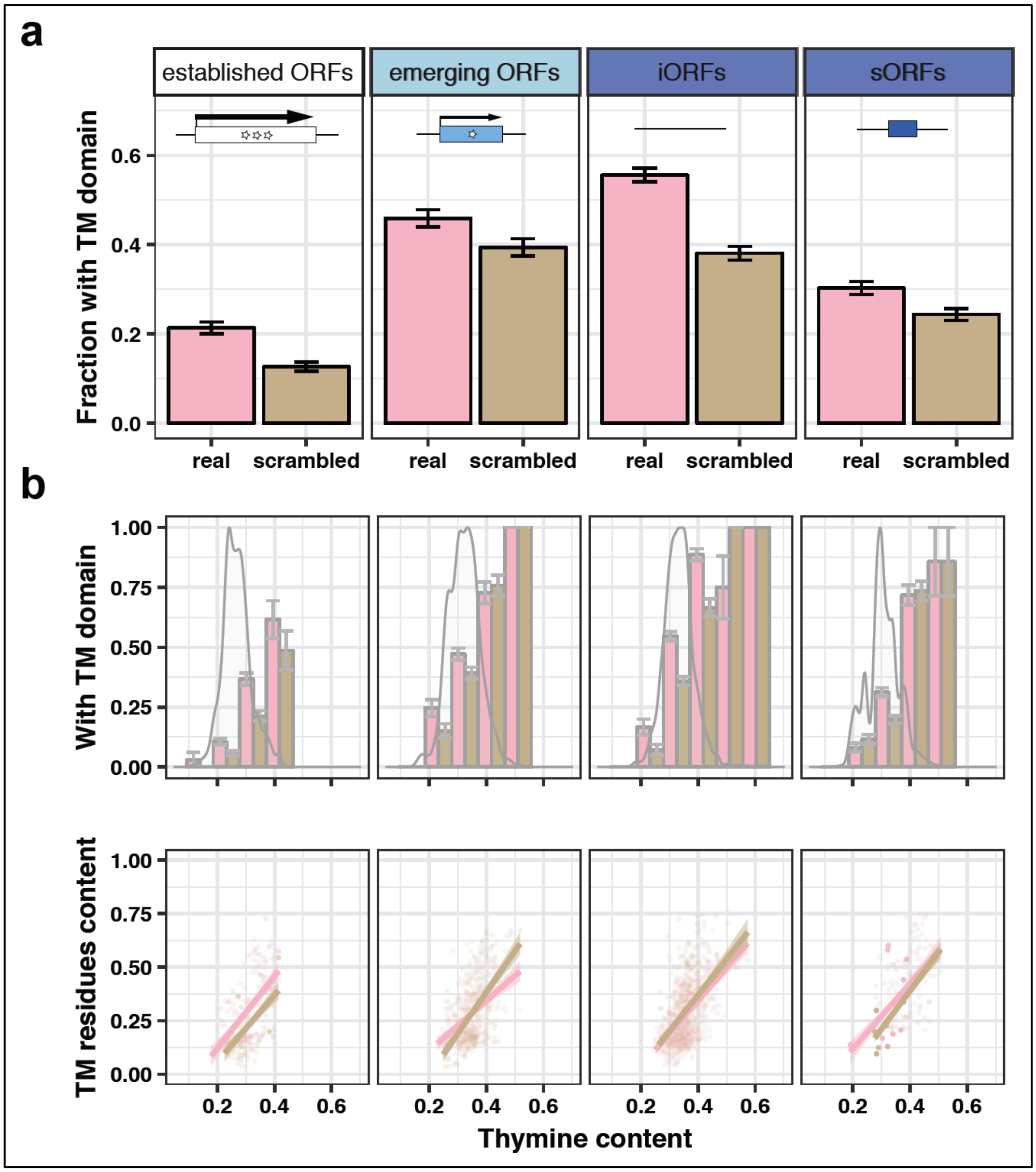

Supplementary Fig. 7. Analysis of TM propensity without sampling to control for length.

a. Propensity of ORFs to form TM domains. Established ORFs, iORFs and sORFs have been sampled (n=1,000) with replacement to follow the length distribution of emerging ORFs. Error bars represent standard error of the proportion.

b. Thymine content influences TM propensity. Top panel: Bar graph represents the fraction of sequences from (**a**) predicted to encode a putative TM domain, binned by thymine content (bin size = 0.1) and compared between real (pink) and scrambled (green) sequences. Error bars represent standard error of the proportion. Overlaid density plots represent the distribution of all sequences in each category. Bottom panel: scatterplot showing the fraction of sequence length predicted to be TM residues as a function of thymine content. Only sequences from (**a**) predicted to encode a TM domain are included in the bottom panel plots. Individual real (pink) and scrambled (brown) sequences are shown in the scatterplot with transparency; points of higher intensity indicate that sequences sampled multiple times. Linear fits with 95% confidence intervals are shown.

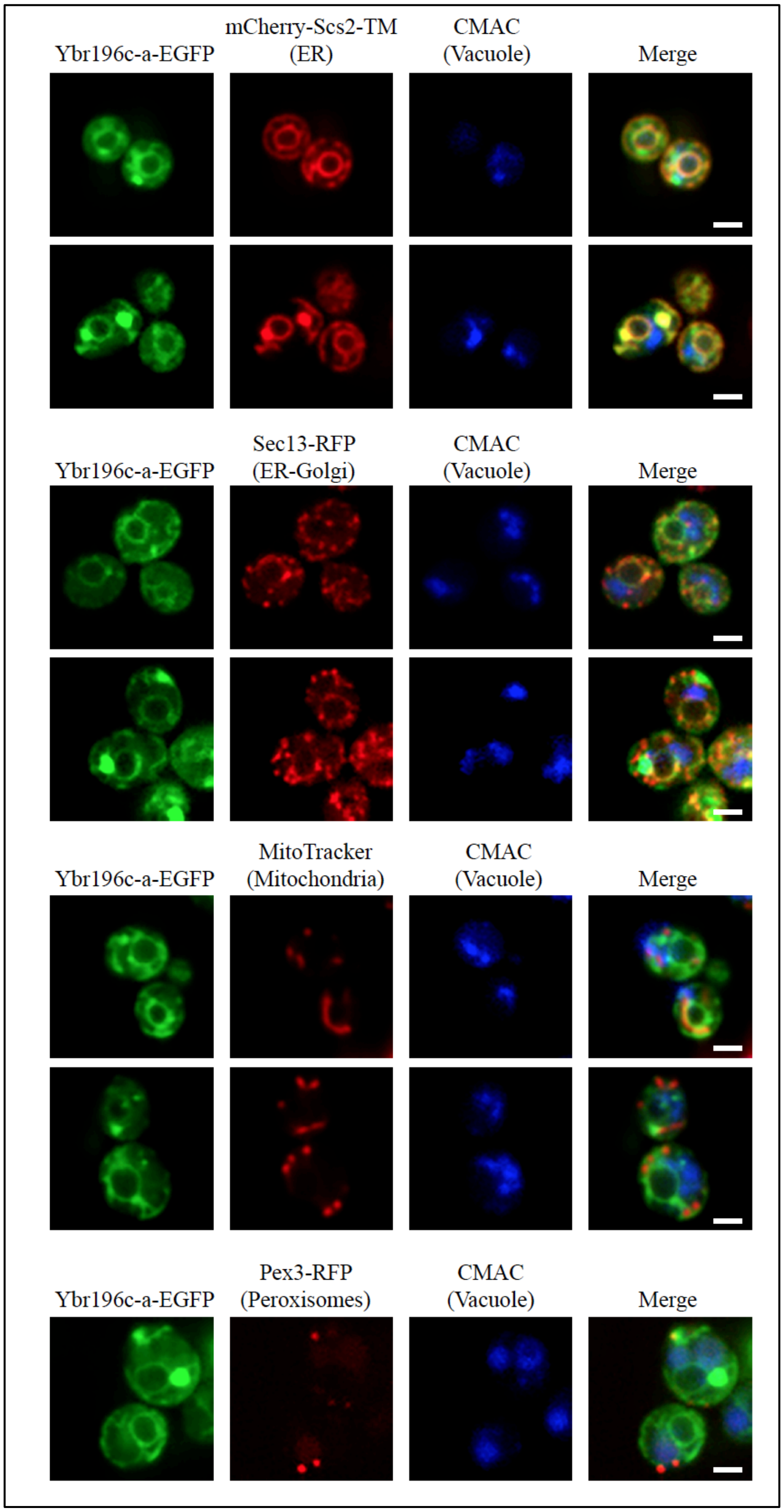

Supplementary Fig. 8. Co-localization studies with Ybr196c-a-EGFP.

From top to bottom, plasmid-borne Ybr196c-a-EGFP and CMAC blue (to mark vacuoles) are visualized with markers of the ER (Scs2p), ER-Golgi (Sec13p), mitochondria (Mitotracker) and peroxisomes (Pex3p). Images were acquired using confocal microscopy and representative micrographs are shown. White line is scale bar = 2µ..

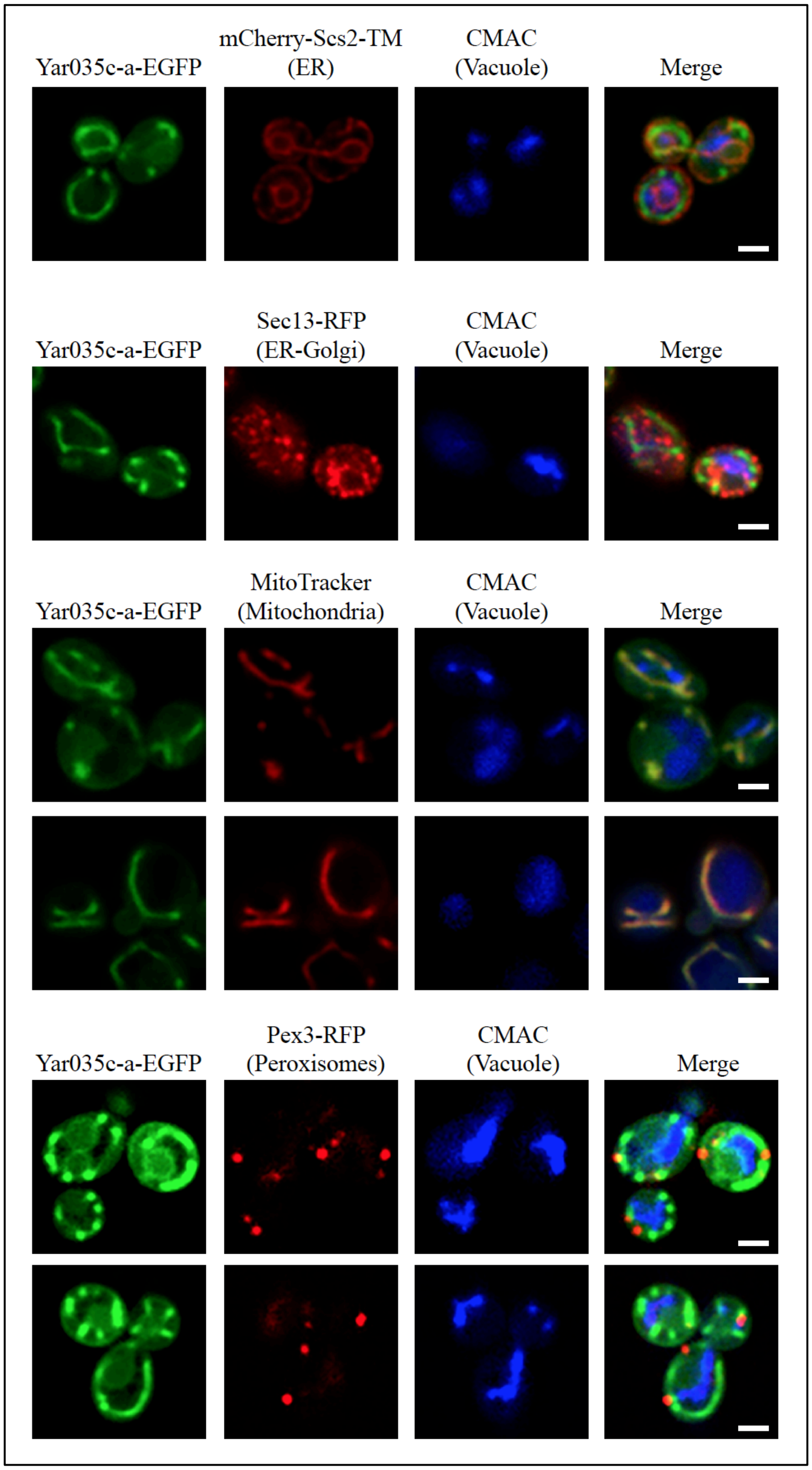

Supplementary Fig. 9. Co-localization studies with Yar035c-a-EGFP.

From top to bottom, plasmid-borne Yar035c-a-EGFP and CMAC blue (to mark vacuoles) are visualized with markers of the ER (Scs2p), ER-Golgi (Sec13p), mitochondria (Mitotracker) and peroxisomes (Pex3p). Images were acquired using confocal microscopy and representative micrographs are shown. White line is scale bar = 2µ..

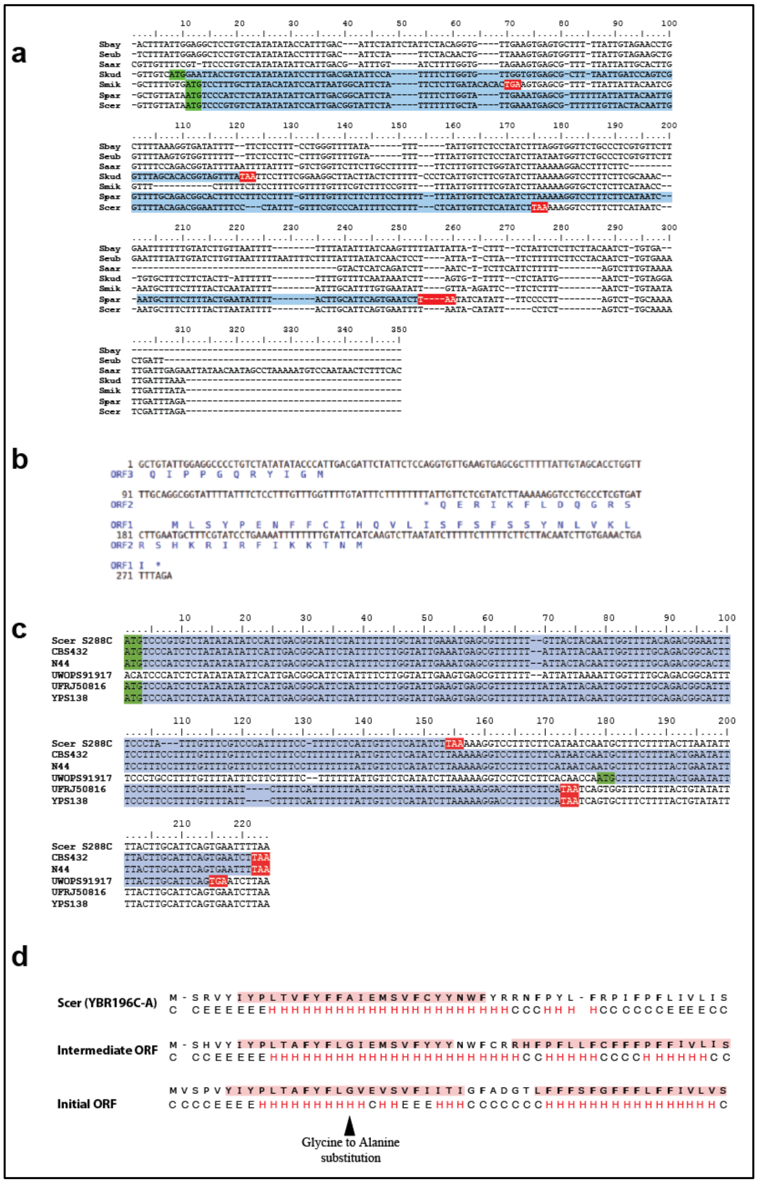

Supplementary Fig. 10. Extant and ancestral homologous regions to *YBR196C-A*.

a. Alignment of homologous regions to *YBR196C-A* in *Saccharomyces* species. Start codons (green), stop codons (red), and ORFs (blue) are highlighted. Alignment was converted to Rich Text Format using BioEdit software (http://www.mbio.ncsu.edu/BioEdit/bioedit.html).

b. ORFs present at the reconstructed ancestral sequence. Translation of ORFs (between a start and a stop codon) longer than 30 nucleotides is shown on the reconstructed ancestral sequence. ORFs identified using NCBI's ORFfinder (https://www.ncbi.nlm.nih.gov/orffinder/).

c. Natural sequence variation in the *S. paradoxus* ortholog of *YBR196C-A*. Start codons (green), stop codons (red), and ORFs (blue) are highlighted. Alignment was converted to Rich Text Format using BioEdit software and subsequently colorized. The *S. cerevisiae* reference sequence is aligned with the syntenic sequence in five *S. paradoxus* isolates.

d. Key sequence changes impair TM helices formation. Alignment between translations of *YBR196C-A* (*S. cerevisiae*), *YBR_Intermediate* and *YBR_Initial* with Phobius predictions highlighted in pink and PsiPred secondary structure predictions shown underneath (H; helix; E: extended strand; C: coil). Arrow indicates helix a breaking residue in the conserved N-terminal predicted TM domain.

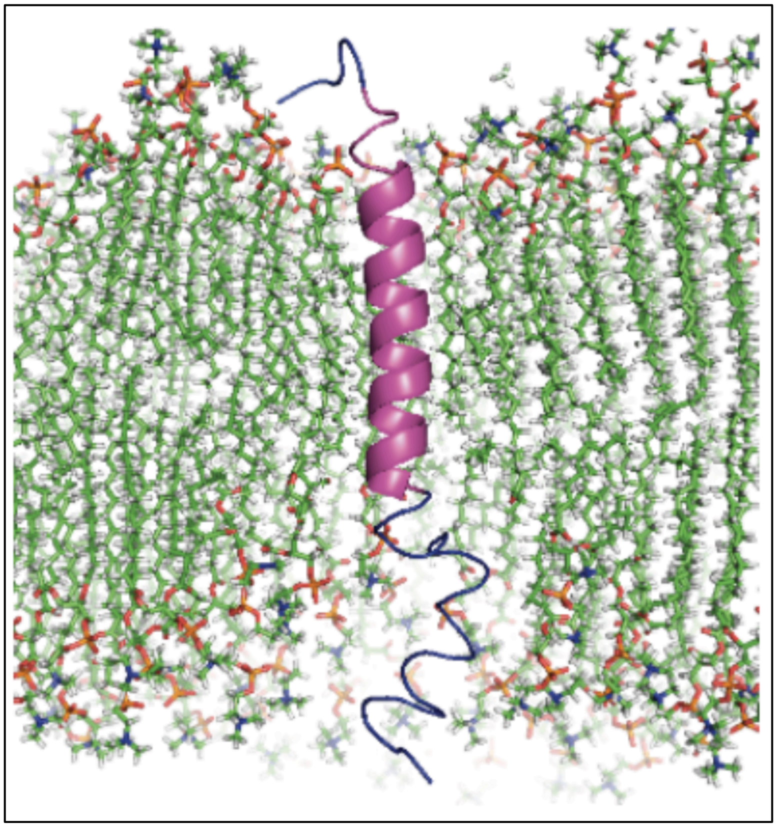

Supplementary Fig. 11. Ybr196c-a is predicted to stably integrate membranes.

Molecular dynamics simulation shows that, after 200ns, the peptide has kept the helix intact, with N and C terminal tails interacting with the surface of the lipid bilayer. Structure model from **Fig. 7d**.

Supplementary Table 1. Yeast growth conditions

**Screening condition (N/C)**	**Complete name**	**Composition**
N/A	SC+GLU+G418+5FOA	Synthetic complete + 2% Glucose + 100μg/mL G418 + 0.01% 5-FOA
N/A	SC+GLU+G418	Synthetic complete + 2% Glucose + 100μg/mL G418
N/A	SC+GAL+G418	Synthetic complete + 2% Galactose + 100μg/mL G418
N/A	SC-URA+GLU+G418	Synthetic complete (-uracil) + 2% Glucose + 100μg/mL G418
+/+	SC-URA+GAL+G418	Synthetic complete (-uracil) + 2% Galactose + 100μg/mL G418
+/++	SC-URA+GAL+RAF+G418	Synthetic complete (-uracil) + 2% Galactose + 1% Raffinose + 100μg/mL G418
++/+	SC-URA+CASE+GAL+G418	Synthetic complete (-uracil) + casamino acids + 2% Galactose + 100μg/mL G418
++/++	SC-URA+CASE+GAL+RAF+G418	Synthetic complete (-uracil) + casamino acids + 2% Galactose + 1% Raffinose + 100μg/mL G418
-/++	SD-URA+GAL+RAF+G418	Synthetic defined (+Methionine, Histidine, Lysine, Leucine) + 2% Galactose + 1% Raffinose + 100μg/mL G418

SC (Synthetic complete) and SD (Synthetic defined) media are composed by 0.175% Yeast Nitrogen Base without amino acids and without ammonium sulfate and supplemented with 0.1% L-Glutamic Acid and 0.2% dropout amino acids. The dropout mixes were made with 10g of Leucine, 3g of Adenine, 0.2g of para-aminobenzoic acid and 2g of the remaining amino acids for each dropout mix.

Supplementary Table 2. Genotypes of yeast strains used in this study.

Strain	Genotype	Source
BY4741	*MATα his3Δ1, leu2Δ0, met15Δ0, ura3Δ0*	Winston et al.[2]
BarFLEX collection	*MATα can1Δ::STE2pr –S.p.HIS5, lup1Δ::STE3pr-LEU2, his3Δ1, leu2Δ0, ura3Δ0, met15Δ0 ho::KanMX, pGAL-ORF-URA3*	Douglas et al.[1]
ARC0000 (Reference Strain)	*MATα can1Δ::STE2pr –S.p.HIS5, lup1Δ::STE3pr-LEU2, his3Δ1, leu2Δ0, ura3Δ0, met15Δ0 ho::KanMX, pBY011*	This study
ARC0011	*MATα can1Δ::STE2pr –S.p.HIS5, lup1Δ::STE3pr-LEU2, his3Δ1, leu2Δ0, ura3Δ0, met15Δ0 ho::KANMX* *Trp1::P-GPDmCherry-Scs2TM-NatMX*	This study
SEC13-RFP	*MATα his3Δ1 leu2Δ0 lys2Δ0 ura3Δ0 SEC13::RFP kanMX4*	Huh et al.[3]
PEX3-RFP	*MATα his3Δ1 leu2Δ0 lys2Δ0 ura3Δ0 PEX3::RFP kanMX4*	Huh et al.[3]

Supplementary Table 3. Plasmids used in this study.

Plasmid	Description	Source
pBY011	Yeast Gateway expression vector with ARS1, CEN4, Gal1-10 promoter (URA3)	Dana-Farber/Harvard Cancer Center
pDONR223	Yeast Gateway donor vector	Rual et al.[4]
pDONR223-*YBR196C-A*	Entry Clone created by BP recombinase between the donor vector pDONR223 and the ORF *YBR196C-A*	This study
pDONR223-*YAR035C-A*	Entry Clone created by BP recombinase between the donor vector pDONR223 and the ORF YAR035C-A	This study
pDONR223-*YBR_Initial*	Entry Clone created by BP recombinase between the donor vector pDONR223 and the ORF *YBR_Initial*	This study
pDONR223-*YBR_Intermediate*	Entry Clone created by BP recombinase between the donor vector pDONR223 and the ORF *YBR_Intermediate*	This study
pDONR223-*YBR_paradoxus*	Entry Clone created by BP recombinase between the donor vector pDONR223 and the *YBR196C-A S. paradoxus* homologue	This study
pDONR223-*YBR_mikatae*	Entry Clone created by BP recombinase between the donor vector pDONR223 and the *YBR196C-A S. mikatae* homologue	This study
pDONR223-*YBR_ kudryiavzevii*	Entry Clone created by BP recombinase between the donor vector pDONR223 and the *YBR196C-A S. kudryiavzevii* homologue	This study
pAG426GAL-ccdB-EGFP	Yeast Gateway expression vector with 2μ, Gal1 promoter, EGFP (URA3)	Alberti et al.[5]
pAG426GAL-*YBR196C-A*-EGFP	Expression vector created by LR recombination between the destination vector pAG426GAL-ccdB-EGFP and Entry Clone pDONR223-*YBR196C-A*	This study
pAG426GAL-*YAR035C-A*-EGFP	Expression vector created by LR recombination between the destination vector pAG426GAL-	This study

	ccdB-EGFP and Entry Clone pDONR223-*YAR035C-A*	
pAG426GAL-*YBR_Initial*-EGFP	Expression vector created by LR recombination between the destination vector pAG426GAL-ccdB-EGFP and Entry Clone pDONR223-*YBR_Initial*	This study
pAG426GAL-*YBR_Iintermediate*-EGFP	Expression vector created by LR recombination between the destination vector pAG426GAL-ccdB-EGFP and Entry Clone pDONR223-YBR_I*ntermediate*	This study
pAG426GAL-*YBR_paradoxus*-EGFP	Expression vector created by LR recombination between the destination vector pAG426GAL-ccdB-EGFP and Entry Clone pDONR223-*YBR_paradoxus*	This study
pAG426GAL-*YBR_mikatae*-EGFP	Expression vector created by LR recombination between the destination vector pAG426GAL-ccdB-EGFP and Entry Clone pDONR223-*YBR_mikatae*	This study
pAG426GAL-*YBR_ kudryiavzevii* -EGFP	Expression vector created by LR recombination between the destination vector pAG426GAL-ccdB-EGFP and Entry Clone pDONR223-*YBR_ kudryiavzevii*	This study
pSM3149	p404 TRP P-GPDmCherry-Scs2TM-NatMX	Zhou et al.[6]

Supplementary references

1. Douglas, A.C. *et al.* Functional analysis with a barcoder yeast gene overexpression system. *G3 (Bethesda)* **2**, 1279-1289 (2012).
2. Winston, F., Dollard, C. & Ricupero-Hovasse, S.L. Construction of a set of convenient Saccharomyces cerevisiae strains that are isogenic to S288C. *Yeast* **11**, 53-55 (1995).
3. Huh, W.K. *et al.* Global analysis of protein localization in budding yeast. *Nature* **425**, 686-691 (2003).
4. Rual, J.F. *et al.* Human ORFeome version 1.1: a platform for reverse proteomics. *Genome Res* **14**, 2128-2135 (2004).
5. Alberti, S., Gitler, A.D. & Lindquist, S. A suite of Gateway cloning vectors for high-throughput genetic analysis in Saccharomyces cerevisiae. *Yeast* **24**, 913-919 (2007).
6. Zhou, C. *et al.* Organelle-based aggregation and retention of damaged proteins in asymmetrically dividing cells. *Cell* **159**, 530-542 (2014).
