# Supplementary material for: De novo emergence of adaptive membrane proteins from thymine-rich genomic sequences
Source: Nat Commun. 2020 Feb 7;11:781. doi: 10.1038/s41467-020-14500-z (PMC7005711; doi:10.1038/s41467-020-14500-z)
Supplement: Supplementary file 8 — Description of Additional Supplementary Files [file 41467_2020_14500_MOESM8_ESM.pdf]

Description of Additional Supplementary Files

File Name: Supplementary Data 1
Description: Description of yeast ORFs analyzed in this study.

File Name: Supplementary Data 2
Description: Normalized colony sizes of overexpression strains in SCURA+GAL.

File Name: Supplementary Data 3
Description: Results of overexpression screens in 5 environments. This data is visualized in Fig. 3d and then used to label ORFs as adaptive, neutral or deleterious throughout the rest of the manuscript. Scripts to go from this data to the figures panels are available : https://github.com/annerux/AdaptiveTMprotogenes. Column names: hours: time when colony size measurement was taken; n: number of replicates colonies per ORF; normalized_cs: normalized colony size, q_cs: q value; effect_cs: relative fitness category; exp_id: experiment number; exp_environment: experimental environment.

File Name: Supplementary Data 4
Description: Data used to examine genome wide TM propensity in Figs 5a,b and S7a,b. Scripts to go from this data to the figures panels are available : https://github.com/annerux/AdaptiveTMproto-genes. Column names: length: protein sequence length; no_res_TM_Phob: number of residues predicted to be TM by Phobius; no_hel_Phob: number of TM helices predicted by Phobius; type: type of ORF; mode: mode of ORF (scrambled, real); T: thymine percentage.

File Name: Supplementary Data 5
Description: Data on small ORFs and the corresponding intergenic regions, used in Fig. 5c. Scripts to go from this data to the figures panels are available : https://github.com/annerux/AdaptiveTMproto-genes. Column names: nameNGO: name of the ORF; nameINTER: name of the intergenic region; INTER_length_nt: size of the intergenic region; NGO_length_aa: length of product of ORF; NGO_no_hel: number of predicted TM helices; fract_lengths: fraction of the intergene occupied by the ORF; bin: bin of the fraction of intergene.
