# Supplementary material for: De novo emergence of adaptive membrane proteins from thymine-rich genomic sequences
Source: Nat Commun. 2020 Feb 7;11:781. doi: 10.1038/s41467-020-14500-z (PMC7005711; doi:10.1038/s41467-020-14500-z)
Supplement: Supplementary file 9 — Reporting Summary [file 41467_2020_14500_MOESM9_ESM.pdf]

natureresearch

Corresponding author(s): Anne-Ruxandra Carvunis

Last updated by author(s): 19/12/19

Reporting Summary

Nature Research wishes to improve the reproducibility of the work that we publish. This form provides structure for consistency and transparency in reporting. For further information on Nature Research policies, see Authors & Referees and the Editorial Policy Checklist.

Statistics

For all statistical analyses, confirm that the following items are present in the figure legend, table legend, main text, or Methods section.

n/a	Confirmed	
☐	☒	The exact sample size (*n*) for each experimental group/condition, given as a discrete number and unit of measurement
☐	☒	A statement on whether measurements were taken from distinct samples or whether the same sample was measured repeatedly
☐	☒	The statistical test(s) used AND whether they are one- or two-sided<br>*Only common tests should be described solely by name; describe more complex techniques in the Methods section.*
☒	☐	A description of all covariates tested
☐	☒	A description of any assumptions or corrections, such as tests of normality and adjustment for multiple comparisons
☐	☒	A full description of the statistical parameters including central tendency (e.g. means) or other basic estimates (e.g. regression coefficient) AND variation (e.g. standard deviation) or associated estimates of uncertainty (e.g. confidence intervals)
☐	☒	For null hypothesis testing, the test statistic (e.g. *F*, *t*, *r*) with confidence intervals, effect sizes, degrees of freedom and *P* value noted<br>*Give P values as exact values whenever suitable.*
☒	☐	For Bayesian analysis, information on the choice of priors and Markov chain Monte Carlo settings
☒	☐	For hierarchical and complex designs, identification of the appropriate level for tests and full reporting of outcomes
☐	☒	Estimates of effect sizes (e.g. Cohen's *d*, Pearson's *r*), indicating how they were calculated

Our web collection on statistics for biologists contains articles on many of the points above.

Software and code

Policy information about availability of computer code

Data collection	Colony image processing and relative fitness estimations were performed with custom scripts available at https://github.com/bbhsu/protogene-analysis.
Data analysis	bedtools subtract v 2.23.0 : extraction of non-annotated genomic regions<br>MUSCLE : multiple alignment of syntenic blocks<br>TMHMM server v 2.0 : prediction of transmembrane domains<br>Phobius server : prediction of transmembrane domains<br>Robeta : protein 3D structure prediction<br>CHARMM-GUI : molecular dynamics simulations<br>Visual Molecular Dynamics : analysis and vizualisation of molecular trajectories<br>PyMol : analysis and vizualisation of molecular trajectories<br>NCBI TBLASTN & BLASTP v 2.6.0+ : sequence similarity searches<br>MACSE v1 : codon-aware sequence alignment<br>PHYML v 20120412 : phylogenetic reconstruction<br>PRANK v 140603: ancestral sequence reconstruction<br>PAML v 4.8 : testing of evolutionary hypothesis<br>Nikon NIS-Elements : image acquisition parameters manipulation<br>ImageJ, Photoshop : image processing<br><br>Scripts for synteny analysis are available at https://github.com/oacar/synal<br>Scripts for all other analyses are available at https://github.com/annerux/AdaptiveTMproto-genes

For manuscripts utilizing custom algorithms or software that are central to the research but not yet described in published literature, software must be made available to editors/reviewers. We strongly encourage code deposition in a community repository (e.g. GitHub). See the Nature Research guidelines for submitting code & software for further information.

Data

Policy information about availability of data

All manuscripts must include a data availability statement. This statement should provide the following information, where applicable:

- Accession codes, unique identifiers, or web links for publicly available datasets
- A list of figures that have associated raw data
- A description of any restrictions on data availability

All data generated/analyzed in this study are available in the main text, in the Supplementary figures and tables and as Supplementary Data files. Supplementary Data files contain raw data related to figures 2,3,4,5, S1, S3, S4, S5, S6, S7. All supplementary data are also on github: https://github.com/annerux/AdaptiveTMproto-genes. The source data underlying Figure 6c,d are provided as a Source Data file. Strains are available from the corresponding authors upon reasonable request.

Field-specific reporting

Please select the one below that is the best fit for your research. If you are not sure, read the appropriate sections before making your selection.

☒ Life sciences ☐ Behavioural & social sciences ☐ Ecological, evolutionary & environmental sciences

For a reference copy of the document with all sections, see nature.com/documents/nr-reporting-summary-flat.pdf

Life sciences study design

All studies must disclose on these points even when the disclosure is negative.

Sample size	All annotated yeast ORFs were included.
Data exclusions	Overlapping ORFs were excluded from the analysis of selected effects since it would be impossible to discern which of the overlapping genes is the cause of the effect.
Replication	Thousands of technical replicates were included in the overexpression assays to boost robustness of the analysis . Replicates were also performed for the microscopy analyses, within which multiple cells were examined.
Randomization	A randomization procedure was followed to empirically calculate the expected fraction of emerging ORFs that would be found to increase relative fitness in more than one environment under a stochastic null model where ORFs are drawn randomly from the set of emerging ORFs never found deleterious in our experiments. Five draws with replacement of the same number of elements as the real data were performed 10,000 times.
Blinding	Blinding was not relevant in this study since the hypotheses tested depended on already established groups of genes

Reporting for specific materials, systems and methods

We require information from authors about some types of materials, experimental systems and methods used in many studies. Here, indicate whether each material, system or method listed is relevant to your study. If you are not sure if a list item applies to your research, read the appropriate section before selecting a response.

Materials & experimental systems

n/a	Involved in the study
☒	☐ Antibodies
☒	☐ Eukaryotic cell lines
☒	☐ Palaeontology
☐	☒ Animals and other organisms
☒	☐ Human research participants
☒	☐ Clinical data

Methods

n/a	Involved in the study
☒	☐ ChIP-seq
☒	☐ Flow cytometry
☒	☐ MRI-based neuroimaging

Animals and other organisms

Policy information about studies involving animals; ARRIVE guidelines recommended for reporting animal research

Laboratory animals	The study did not involve laboratory animals, only various strains of S. cerevisiae.
Wild animals	Not applicable
Field-collected samples	Not applicable

Ethics oversight | No ethical approval guidance was necessary

Note that full information on the approval of the study protocol must also be provided in the manuscript.
